# Aggression towards the GP: can we profile the GP–victim? A cross-section survey among GPs

**DOI:** 10.3399/bjgpopen18X101604

**Published:** 2018-09-19

**Authors:** Vince Demeur, Sarah Devos, Esther Jans, Birgitte Schoenmakers

**Affiliations:** 1 Student Researcher, Department of Public Health and Primary Care, Academic Centre of General Practice, University of Leuven, Leuven, Belgium; 2 Student Researcher, Department of Public Health and Primary Care, Academic Centre of General Practice, University of Leuven, Leuven, Belgium; 3 Student Researcher, Department of Public Health and Primary Care, Academic Centre of General Practice, University of Leuven, Leuven, Belgium; 4 Professor, Department of Public Health and Primary Care, Academic Centre of General Practice, University of Leuven, Leuven, Belgium

**Keywords:** Aggression, personality, general practitioner, patient

## Abstract

**Background:**

Aggression against GPs has increased in the past decade. Depending on experience, interpretation, and personality, the interpretation of aggressive patient behaviour will differ among doctors.

**Aim:**

To investigate how often GPs experience aggression in a 1-year time span and what the relationship is between the GP's personality (based on the 'Big Five' personality traits) and the reporting of aggression. Secondly, to investigate how personality is related to feeling safe.

**Design & setting:**

Flemish (Belgian Federal State) GPs were questioned in a cross-sectional design by online survey. GPs were recruited and questioned in their professional environment.

**Method:**

Outcome measures were the 'Big Five' personality traits ('reserved' versus 'outgoing', 'compassionate' versus 'challenging', 'efficient' versus 'careless', 'confident' versus 'nervous', and 'cautious' versus 'innovative', based on Cattel's 'Big Five' model of personality), the type of aggression, the reporting of aggression, and feeling safe.

**Results:**

Both (*n* = 247) male and female doctors considered physical contact and verbal intimidation as aggression. Female doctors were more likely to consider sexual harassment as aggression. The majority of GPs were confronted with verbal aggression. More than half considered physical aggression as the most threatening. GPs with 'reserved' and 'careless' personality types were more likely to experience aggression. GPs with 'innovative', 'challenging', or 'confident' personality types were also at increased risk, but to a lesser extent than those with 'reserved' and 'careless' personalities. GPs with 'efficient' and 'innovative' personalities were more likely to report incidents. Male GPs and those with 'efficient' personalities felt safer. GPs with 'confident' and 'cautious' personalities were more likely to feel unsafe.

**Conclusion:**

The results of this study might help future interventions and support strategies (designed to prevent aggressive incidents or help GPs cope with them) to target the vulnerable groups. Further research should therefore explore the results of these data in depth and on a larger sample size.

## How this fits in

Aggression from patients to doctors is increasing. Past research, profiling of the aggressor, led to interventions. By profiling the victim (victim personality and other features), interventions can be tailor-made and target the vulnerable professional.

## Introduction

Aggression against GPs has been a known phenomenon for a long time, but the number of incidents seems to have increased in recent years. Recently, the murder of a GP in Belgium by a patient during a home visit brought attention to this issue again.^1,2^ Although there is a national aggression reporting system, not every doctor who is confronted with aggression will report this as an incident. Goldberg identifies four levels of aggression: verbal aggression, physical aggression, psychological aggression (threat and intimidation), and sexual harrassment.^3^ Depending on the GP’s experience, interpretation, and personality, the attribution of an incident to one of these levels will differ.

A study in the Netherlands shows that 64% of GPs were confronted with verbal aggression in the last year. Compared with physician assistants, GPs report more incidents of threats, intimidation, and discrimination. Nevertheless, the majority (96%) of GPs feels safe during work. This cross-sectional research also demonstrates that young and/or female GPs, GPs on out-of-hours duty, and GPs in urban or suburban areas experience more aggression.^4^

In Germany, researchers observed that more than half of the surveyed physicians had been confronted with a mild or moderate types of aggression, and more than one in 10 had experienced serious aggression. In this study, only 9% had never experienced any aggression during their professional career. Although most female doctors felt safe in their surgery, only one in three felt safe at home visits during out-of-hours duty.^5^ In a French study, physical or verbal aggression was reported by 56% of the GPs. The reporting of incidents was not related to the sex of the reporting victim. In most cases, aggression occured because the doctor refused to issue a prescription.^6^ Five Australian studies show that almost three-quarters of the surveyed physicians had dealt with aggression at during their career.^7–11^ In these studies, the premise that women are more likely to face aggression was not confirmed. However, women are confronted more often with sexually unacceptable behaviour. GPs with longer working days or fewer years of experience face more episodes of aggression.^11^ According to recent research, there is a lasting impact of aggression towards care professionals: a reduction of daily working hours, lower job satisfaction, and an intention to quit the job.^12,13^

Although many researchers focus on profiling the aggressor and describing the context of incidents, there is no consensus on outcome. Additionally, the research outcome seems to disagree with reality: the high prevalence of aggression incidents reported in research is not in accordance with the number of incidents reported in reality. It is not unthinkable that the experiencing and reporting of incidents is influenced by context and circumstances.

This study investigates how often a GPs face aggression, and what the relationship is between the personality of the GP and the type of aggressive incidents. It also investigates how the personality of the GP is related to the feeling of safety.

## Method

### Population

The target population consisted of all Flemish (Belgian Federal State) GPs. GPs were recruited through the local and regional professional registration network of active GPs. There were no exclusion criteria.

### Design

A cross-sectional observational study was quantitatively carried out through an online survey. The survey was developed based on scientific literature and expert consultation. Questions were answered by a yes/no option, by a multiple choice option, or by free text fields. Five personality traits were measured using a 4-point Likert scale: 'reserved' versus 'outgoing', 'compassionate' versus 'challenging', 'efficient' versus 'careless', 'confident' versus 'nervous', and 'cautious' versus 'innovative'. These personality traits were based on Cattel's 'Big Five' model of personality.^14^ This model was developed and refined over the past 2 decades and, in application, is an understandable and comprehensive way to categorise personality traits (Box 1).

**Box 1.** Explanation of 'Big Five' personality traits

Lower score	Personality trait	Higher score
Practical, conventional, prefers routine	Openness	Curious, wide range of interests
Impulsive, careless	Conscientiousness	Hardworking, dependable, organised
Quiet, reserved, withdrawn	Extroversion	Outgoing, warm, seeks adventure
Critical, uncooperative, suspicious	Agreeableness	Helpful, trusting, empathic
Calm, secure	Neuroticism	Anxious, unhappy

The survey also contained demographic and practice characteristics of physicians, self-reported frequency of aggression, characteristics of aggressor, circumstances of aggression, measures taken to prevent aggression, and impact of aggression. In order to gain a better understanding of what GPs consider as aggression, participants were asked to complete each answer with a free-text comment.

The internet survey was sent early December 2016 to all Flemish GP networks. The network coordinators distributed the survey to the network members through a link on the website or by a printed flyer. In March 2017, a reminder was sent to the network coordinator and the link to the survey was announced in the newsletter of Domus Medica (a nonprofit syndical, training, and scientific family medicine organisation) and posted on the website of the ICHO (Interuniversitair Centrum voor Huisartsenopleiding, the Flemish nonprofit organisation of postgraduate training in family medicine). The data collection of the survey took place between 1 December 2016 and 2 April 2017.

### Outcome measures

The outcomes of the study are the prevalence of aggression, the characteristics of the aggression, and the personality characteristics of the physician who experiences aggression or experiences feeling unsafe.

### Data analysis

In order to work with a 95% confidence interval (CI) and a 5% error margin, a sample size of 369 responders was required to generate a power of 0.90. This was calculated on the basis of 9157 licensed GPs and family doctors in training in Flanders in 2015, and the prevalence and distribution of aggression incidents.^15^ The results of the survey were exported from Google Forms to Excel, and univariate analyses were calculated using Excel (version 2016). The cut-off for 'frequently experiencing aggression’ was set at ‘monthly or more often’. The personality characteristics were described on a 4-point Likert-type scale. Responses 1 and 2 were considered as personality X; responses 3 and 4 as the opposite, personality Y. Multivariate regression analyses were carried out with the statistical program SAS (version 9.4), and the correlations between independent variables (sex, age, region, personality trait) and dependent variables (ever experienced aggression, declaration of incident, and feelings of safety) were calculated. The personality characteristics were interpreted as a continuous spectrum on a Likert scale, ranging from more personality X to more personality Y.

## Results

### Sample size and feature responders

Of the 248 completed surveys, one was excluded because of a discrepancy between the age and number of years of experience. The results were based on a sample of 247 GPs. Given this sample size, the error margin was 6.15%.

Most responders were female (60.3%). In addition, 47.4% were aged <35 years. More GPs operated in a rural area (58.7%) than an urban area (41.3%). Most participants (52.6%) worked in a group practice (see Table 1).

**Table 1.** Demographic features of the responders

		Male, *n*	Female, *n*	Total, *n*
Age, years	25–35	24	93	117
	36–45	10	21	31
	46–55	24	24	48
	>55	40	11	51
Experience, years	>40	9	1	10
	31–40	32	7	39
	21–30	23	25	48
	11–20	7	16	23
	6–10	7	18	25
	0–5	12	40	52
	GP resident	8	42	50
Province	Antwerp	15	21	36
	Brussels	0	2	2
	Limburg	9	18	27
	East Flanders	31	41	72
	Flemish Brabant	25	48	73
	West Flanders	18	19	37
Location	Rural	60	85	145
	Urban	38	64	102
Practice	Single-handed	52	29	81
	Two-handed	10	26	36
	Group	36	94	130
Total		98	149	247

There was an equal distribution of personality traits among the responders except for the 'nervous' trait: this personality was largely underrepresented (Table 2).

**Table 2.** Personality traits of the responders, *n *(*N* = 247)

Likert scale	1	2	3	4	
Reserved	17	94	95	41	Outgoing
Compassionate	21	125	93	8	Challenging
Efficient	71	98	68	10	Careless
Confident	93	123	31	0	Nervous
Cautious	20	96	108	23	Innovative

### Prevalence and context of aggression

Both male and female doctors considered physical contact (gripping, striking, or slapping) and verbal intimidation as aggression. In addition, female doctors were more likely to consider sexual harassment or intimidation as aggression than male doctors: 81.9% of female responders considered this as aggression versus only 54.1% of male responders (Table 3).

**Table 3.** Types of aggression, as experienced by GPs. Frequency of GPs experiencing aggression type versus frequency of considering it the most threatening type of agression

Type of aggression	Male, *n*	Female, *n*	Total, *n*
Raising voice	25	62	87
Scolding	72	125	197
Verbal intimidation	79	134	213
Violating privacy	43	66	109
Touching	36	62	98
Grabbing, slapping, kicking	83	135	218
Sexual intimidation	53	122	175
Other	5	8	13
**Confronted with type of aggression/considered as most threatening**			
Physical	22/76	29/81	
Verbal	85/3	137/2	
Psychological	14/12	38/10	
Sexual	2/5	17/53	
Other	2/2	5/3	

Of all responders, 79.8% experienced aggression in the last year. A considerable number (11.3%) experienced aggression every month or more often. Only 5.3% of the GPs reported never having been the victim of aggression. Of the responders, 89.8% had been confronted with verbal aggression, 21.1% with psychological aggression, 20.6% with physical aggression, and 7.7% with sexual aggression. However, 63.6% of GPs considered physical aggression as the most threatening form. While verbal aggression appeared the most common type of aggression, only 1.9% experienced this as the most threatening type (Table 3).

In 40.1% of the cases, the last incident of aggression took place during the consultation, in 22.7% during home visits, and in 18.6% by telephone. Only 6.5% of the incidents occurred in the waiting room. In more than half of the cases (55.1%) the aggressor was known by the doctor. In 32.1% of the incidents, the aggressor had a known psychiatric history, and 11.7% of the aggressors were under influence of alcohol, drugs, or medication. Triggers of aggression were not meeting the patient’s demands (72.5%), disagreement with the proposed treatment (41.7%), and the exceeding of scheduled waiting times (30.8%), as shown in Table 4.

**Table 4.** Context of aggression incident

	Context	Total, *n*
Situation	Consultation	99
	Home visit	56
	Out-of-hours	16
	Public	2
	Telephone	46
	Other	28
Characteristics of aggressor	Known patient	136
	Unknown patient	59
	Under influence of drugs, alcohol, or medication	29
	Psychiatric history	79
	Criminal history	17
	Repeat aggressor	9
	Other	29
Cause	Money	13
	Waiting time	76
	Discussion about fee	10
	Reaction to bad news	9
	Disagrement with diagnosis	33
	Disagrement with treatment	103
	Not meeting demands	179
	Other	37

Although most responders had experienced aggression, most of them were not severely mentally or physically injured. However, 31.2% of physicians reported an emotional impact, 5.7% material damage (infrastructure), and 2.8% physical damage as a consequence. Two GPs experienced temporary medical leave from work. Only 12.6% of the responders reported the incident. Of those who never reported the incident, 47.7% considered doing so in the future. Finally, 88.7% of GPs generally felt safe (Table 5).

**Table 5.** Impact of aggressive incident and frequency of reporting

	Male, *n*	Female, *n*	Total, *n*
Physical injury	3	4	7
Material damage	8	6	14
Emotional damage	25	52	77
Medical leave	2	0	2
Other	3	8	11
None	65	86	151
Reporting of incident	12	19	31

### Multivariate regression analysis

The multivariate regression analysis did not find a significant link between age, sex, or personality and ‘ever experienced aggression’. The same was true for the analysis of age, sex, practice, and location as independent variables and ‘ever experienced aggression’ as a dependent variable. A more 'efficient' personality was significantly more likely to report the incident than a more 'careless' personality (odds ratio [OR] 0.578, 95% CI = 0.358 to 0.931). A more 'cautious' personality was less likely to report than an 'innovative' personality (Table 6).

**Table 6.** Relationship between GPs' characteristics and reporting an aggressive incident, and relationship between GPs' characteristics and feeling unsafe

Characteristics of GP versus reporting incident	Odds ratio	95% CI
Older age	0.978	0.947 to 1.009
Male	1.503	0.606 to 3.730
Reserved	1.151	0.682 to 1.942
Compassionate	1.111	0.588 to 2.100
Efficient	0.578	0.358 to 0.931
Confident	1.212	0.666 to 2.208
Cautious	1.784	1.014 to 3.138
Characteristics versus feeling unsafe		
Older age	1.008	0.968 to 1.050
Male	0.155	0.038 to 0.637
Reserved	0.650	0.366 to 1.155
Compassionate	1.062	0.540 to 2.088
Efficient	0.576	0.334 to 0.992
Confident	2.460	1.232 to 4.910
Cautious	2.444	1.252 to 4.769

In addition, two multivariate analyses were performed with ‘feeling of safety’ as a dependent variable and age, sex, personality characteristics, practice type, and location as independent variables. These analyses showed a highly significant result for sex (with a significance level of *P*<0.01). Men significantly felt safer than women (OR 0.155, 95% CI = 0.038 to 0.637 versus OR 0.162, 95% CI = 0.043 to 0.615). A more 'efficient' personality was positively associated with feeling safe (OR 0.576, 95% CI = 0.334 to 0.992). 'Confident' personalities were significantly more likely to feel unsafe than 'nervous' personalities (OR 2.460, 95% CI = 1.232 to 4.910); likewise, 'cautious' personalities were significantly more likely to feel unsafe than 'innovative' personalities (OR 2.444, 95% CI = 1.252 to 4.769), as shown in Table 6.

## Discussion

### Summary

This is the first study to explore the relationship between aggression and victim characteristics. Most participating GPs had already been confronted with aggression during their career. Of the responders, 79.8% reported an incident in the last year, and more than one in 10 experienced aggression monthly. The analysis of the personality characteristics showed that GPs with a rather 'reserved' or 'careless' personality type were more likely to face aggression. GPs with 'innovative', 'challenging', or 'confident' personality types were at increased risk, but to a lesser extent than the first category.

### Strengths and limitations

First, the participants were mainly young doctors and women, which might have caused a selection bias. It can be assumed that these doctors participated because they frequently experienced aggression. Second, the survey only addressed aggression incidents during the last 12 months. Third, the response rate of this survey is too low to unconditionally extrapolate the results; based on the sample size of 247 GPs, the error margin is 6.15%. On the other hand, the results of the multivariate analyses were based on meaningful, reliable, and valid models.

### Comparison with existing literature

The results of incidence and prevalence of this study were comparable to those of other studies.^4^–^7^ 

Female doctors were more often confronted with aggression than males. In addition, younger doctors and urban practitioners seemed to be victims more often than older colleagues or rural practitioners. The latter may be related to the higher prevalence of crime in urban areas.^16^ A Dutch national baseline measure of aggressive incidents in general practice published in 2014 also confirmed these groups as most vulnerable to aggression.^4^ Although an earlier Australian study^10^ observed that female and younger GPs, and those working in socioeconomically disadvantaged areas, experienced more aggression, a recent Australian study^11^ did not confirm this. A French study did not observe a significant difference in prevalence of aggression by sex.^6^

Female GPs also reported a lower sense of safety during their work than their male colleagues. A German study confirmed this observation but the difference with their male colleagues was smaller than in the present study.^5^ This difference might be explained by the more frail physiognomy of women as compared to men.^17^ In general, female doctors will be less able to defend themselves in case of a physically aggressive attack than male doctors. Women may, in this case, benefit from training in self-defense and verbal assertiveness.

Men and women also respond in a different way to stressful situations. Female doctors are more likely to suffer emotional damage as a result of aggression than their male colleagues.^17^ In this study, one in three women reported emotional damage compared to one in four men. Women could therefore benefit more than men from interventions to prevent aggression.

Verbal aggression was by far the most common type of aggression in daily practice.^4–11^ Verbal aggression creates an uncomfortable work environment and can affect the psychological wellbeing of the recipient. Communication training in role-playing or in real life situations could improve the interaction with patients and probably reduce triggers leading to aggressive incidents.

About one in five GPs in this study had experienced psychological or physical aggression, which is consistent with other findings.^5–6,18^ As in other studies, only half of the male doctors experienced sexual harassment, compared to more than four in five of all female doctors.^5,11^ Sexual harassment remains a widespread problem in society. Media campaigns aiming to increase public awareness can reduce the incidents of sexual harassment.

Aggression occurred most often during the consultation, though one in five doctors experienced aggression during a home visit. Of course, most patient contacts take place in the doctors’ surgery.^5,19^ Besides this, it is assumed that patients feel more at ease at home, which lowers the risk of discussion leading to aggression. Home visits remain a valuable way of caring for patients. Visiting patients under the supervision of a guard is not commonly accepted or desirable, but might be considered in certain circumstances (for example, unsafe neighborhoods, unknown patients, patients with addiction issues, or night shifts).

In this study, the relationship between aggression by a patient and the characteristics of the GP was also examined. This arose from the observation that prevalence of aggression strongly varies among studies. In addition, although the definition of aggression is well described, the perception or interpretation of aggressive behaviour seems to depend on the victim (it is the eye of the beholder).

GPs with a 'reserved' personality type are perhaps more likely than ‘outgoing’ personalities to interpret patients’ behaviour as aggressive; the survey was based on the subjective interpretation of aggression and not on an objectively defined definition of aggressive behaviour. In addition, it is possible that the physician with a ‘careless’ personality type adheres less to the time schedule, meaning longer waiting times for patients. A long waiting time is identified as a trigger of aggressive patient behaviour. Finally, it is surprising that the doctors with ‘confident’ personality types experienced more aggression than their ‘nervous’ counterparts. This can possibly be explained by the very low number of doctors describing themselves as ‘nervous’.

‘Efficient’ type personalities appeared to feel safer. Those with ‘confident’ and ‘cautious’ type personalities were more likely to report feeling unsafe. Although surprising that 'confident' doctors reported feeling more unsafe than their 'nervous' counterparts, it might be that they are more aware of the real risk of an aggressive incident, and that this makes them feel more unsafe. In addition, 'confident' doctors are less likely to report an aggressive incident and are therefore also less likely to be supported after an incident, which may contribute to feeling unsafe. Remarkably, physicians with ‘efficient’ type personalities who experienced aggression were more likely to report aggression incidents than their ‘cautious’ colleagues. This might suggest that reporting incidents increases the feelings of safety.

### Implications for research

Aggression towards GPs is remarkably common. In the last year, no fewer than eight out of 10 GPs experienced aggression, mostly verbal aggression. The aggressor is usually a known patient. GPs are not used to reporting aggressive incidents so the real prevalence will be higher.^15^

More than one in 10 GPs do not feel safe during their professional hours. In particular female GPs, and GPs with ‘confident’ personality types and ‘cautious’ personality types feel significantly less safe. GPs with a more ‘efficient’ personality type generally feel safer.

The results of this study might help future interventions and support strategies, designed to prevent aggressive incidents or help GPs cope with them, target the vulnerable groups. Since personality traits or features such as sex are unchangeable, interventions should focus on the most efficient and suitable coping strategy. Further research should therefore explore the results of these data in depth and on a larger sample size.
